# Sedentary behavior among Spanish children and adolescents: findings from the ANIBES study

**DOI:** 10.1186/s12889-017-4026-0

**Published:** 2017-01-19

**Authors:** Juan Mielgo-Ayuso, Raquel Aparicio-Ugarriza, Adrian Castillo, Emma Ruiz, Jose M. Avila, Javier Aranceta-Bartrina, Angel Gil, Rosa M. Ortega, Lluis Serra-Majem, Gregorio Varela-Moreiras, Marcela González-Gross

**Affiliations:** 1 ImFINE Research Group, Department of Health and Human Performance, Technical University of Madrid, Madrid, Spain; 2 Spanish Nutrition Foundation (FEN), Madrid, Spain; 3 Department of Preventive Medicine and Public Health, University of Navarra, Pamplona, Spain; 4 CIBER: CB12/03/30038 Fisiopatología de la Obesidad y la Nutrición, CIBERobn, Instituto de Salud Carlos III (ISCIII), Madrid, Spain; 5 Department of Biochemistry and Molecular Biology II, and Institute of Nutrition and Food Sciences, University of Granada, Granada, Spain; 6 Department of Nutrition, Faculty of Pharmacy, Complutense University Madrid, Madrid, Spain; 7 Research Institute of Biomedical and Health Sciences, Department of Health Sciences, University of Las Palmas de Gran Canaria, Las Palmas de Gran Canaria, Spain; 8 Department of Pharmaceutical and Health Sciences, Faculty of Pharmacy, CEU San Pablo University, Madrid, Spain

**Keywords:** Sedentary lifestyle, Physical activity, Youth, Child, ANIBES Study

## Abstract

**Background:**

An increase of sedentary behaviors far from the Mediterranean lifestyle is happening in spite of the impact on health. The aims of this study were to describe sedentary behaviors in children and adolescents.

**Methods:**

A representative sample of 424 Spanish children and adolescents (38% females) involved in the ANIBES study was analyzed regarding their sedentary behaviors, together with the availability of televisions, computers, and consoles by means of the HELENA sedentary behavior questionnaire.

**Results:**

For the total sample of children, 49.3% during weekdays and 84% during weekends did not meet the recommendation of less than 2 hours of screen viewing per day. The use of TV was higher during weekdays (*p* < 0.05) and there were significant differences between adolescents and children (16.9 vs. 25.1%, *p* < 0.05). The use of computer, console games and of internet for non-study reasons was higher during weekends (*p* < 0.001). Adolescents played more computer games and used more internet for non-study reasons than children during both weekdays and weekends (*p* < 0.05 and *p* < 0.001, respectively). The use of internet for academic reasons was lower in children (*p* < 0.001) than adolescents during weekends; however, no significant differences were found between sexes. In addition, more than 30% of the children and adolescents had at least one electronic device in their bedrooms.

**Conclusions:**

Spanish children and adolescents are not meeting the recommendations regarding the maximum of screen viewing (<2 h/day), especially during the weekend, for all of sedentary behaviors. Urgent strategies and intervention studies are needed to reduce sedentary behavior in young people.

## Background

Sedentary lifestyle is of increasing concern around for decades in European countries and worldwide [1]. In adults, it has been identified as an independent risk factor for chronic diseases [2]. In children and adolescents, results are more controversial [3–9]. Results from cross-sectional studies indicate that young people who are even slightly sedentary may have greater fat mass, higher BMI, and increased risk of being overweight or obese, independently of their physical activity (PA) [10]. On the contrary, a recent systematic review analyzing the prospective relationship of sedentary behaviors and health outcomes concluded that current evidence is unconvincing [11]. Other authors have proposed that it is not the general sedentary behavior, but the increased use of new technologies, particularly watching television, playing digital games, and using computers that could be critical [12]. This so called screen-based sedentary behavior has been associated with a range of adverse health consequences [12, 13].even independently of their impact on PA [14], such as adiposity, aerobic fitness, quality of life, self-esteem, pro-social behavior, academic achievement, depression and anxiety [15]. Other authors conclude that there is not enough data available to draw conclusions on this issue, specifically if children and adolescents are sedentary but active at the same time [16].

In fact, regular PA is recommended for improvement of overall health and to facilitate weight control [17, 18]. Among young people, PA also fosters optimal physical and cognitive growth and development [14, 19]. Therefore, children and adolescents aged 5 to 17 years should accumulate at least 60 min of moderate- to vigorous-intensity PA daily [20]. In a recent report from the ANIBES study, we observed that a total of 55.4% of Spanish children and adolescents are not meeting these international PA recommendations [21]. More recently, apart from meeting PA guidelines, reducing sedentary behavior has become an additional goal in public health [10], even if the above-mentioned controversy exists.

With the aim of reducing screen time among children and adolescents, the American Academy of Pediatrics (AAP) in 2001 had already recommended that young people should not spend more than 2 h per day on screen viewing [22], although these 2-h cut-off are controversial and families are confused for good reason [23, 24], as just discussed above. Other countries also launched screen-time recommendations for children and adolescents more recently [25]. At a global level, data are inconsistent regarding time spent on sedentary behaviors. In some European countries, 61% of children aged between 11 and 15 years watched TV more than 2 h per day. In adolescents from the HELENA study, 58% of males and 53% of females watched TV more than 2 h per day during weekend days [26]. The publication of these data raised the awareness that adolescents living in Europe are probably not meeting recommendations regarding time spent in front of a screen, especially during weekends. Moreover, the absence of a TV in the adolescents’ bedrooms has been identified as a protective factor [26]. Regardless of the possible negative consequences for health in early life, sedentary behaviors seem to track from childhood to adolescence and into adulthood [27].

To the best of our knowledge, the prevalence of sedentary screen based behaviors among children and adolescents living in Spain is not well known currently. In order to direct policies that promote interesting activities in different settings (family, school, community) for reducing sedentary screen behaviors, we hypothesized that in Spain as happens in other European countries, there is a high degree of children and adolescents who have a high use of screen devices. There could be a sex (gender) and age effects on this behavior that should be considered in public health, concretely that boys and adolescents the most users of screen devices. The ANIBES study allows us to approach this hypothesis by describing the prevalence of sedentary behaviors in a representative sample of children and adolescents from Spain. In particular, the objectives of this study were: (1) to describe sedentary behaviors by age and sex, (2) to describe electronic device availability at home (TV, computer and console), and (3) to examine the determinants for excess (≥2 h/day) screen time in total and by individual device.

## Methods

### Study design and participants

The design, protocol, and methodology of the ANIBES Study have been already described in detail elsewhere [28, 29]. Briefly, the ANIBES Study was designed to carry out an accurate updating of PA patterns, energy expenditure, food and beverage intake, dietary habits/behavior and anthropometric data of the Spanish population (9–75 years, *n* = 2009).

The participants were selected from seven areas (Northeast, East, South, West, North Central, Metropolitan Barcelona and Madrid) and the Canary Islands, in municipalities of at least 2000 inhabitants. It was conducted through a stratified multistage sampling with 128 sampling points all over Spain, in order to guarantee more coverage and representativeness. No previous pre-recruitment was considered in order to minimize the risk of bias in responses. The present paper is focused on children and adolescents (9–17 years, *n* = 424, considering two age groups: children (9–12 years, *n* = 213; 40.8% girls) and adolescents (13–17 years, *n* = 211; 35.1% girls) Several exclusion criteria were applied: following a therapeutic diet due to a recent surgery or taking any prescription medicine; suffering a transitory pathology (i.e., flu, gastroenteritis, chickenpox, etc.) at the time of the fieldwork. However, individuals under the following conditions were considered eligible to be included: those following dietary advice such as for prevention of diabetes, with diagnosed allergy and/or food intolerance, or those suffering a metabolic disease such as hyperthyroidism or hypothyroidism [28, 29].

All participants were informed of the protocol and risks/benefits and all adults signed a written consent form prior to participation. In the same line, informed written consent from children and adolescents was obtained from participants and parents or guardians. The final protocol was approved by the Ethics Committee for Clinical Research of the Region of Madrid, Spain [28, 29]. Fieldwork for the ANIBES study took place from mid-September 2013 to mid-November 2013 (2 months), and two previous pilot studies were scheduled (in June and September, 2013).

### Data collection

Patterns of sedentary behavior were assessed using the HELENA (Healthy Lifestyle in Europe by Nutrition in Adolescence) sedentary behavior questionnaire [26]. This questionnaire displayed moderate seven-day test-retest reliability (intraclass correlation coefficients ranging from 0.36 to 0.77, and 0.71 to 0.78 for weekdays and weekends, respectively) when assessing the sedentary patterns in a sub-sample of 183 adolescents aged 13 to 18 years from the HELENA study.

Children and adolescents reported for both studies the hours of TV viewing, playing with computer games, playing with console games, surfing the internet for non-study reasons, surfing the internet due to study reasons, and studying (outside school hours) during week and weekend days. They selected one of the following categories: (1) none, (2) less than ½ hour, (3) between ½–1 h, (4) between 1 and 2 h, (5) between 2 and 3 h, (6) between 3 and 4 h, (7) more than 4 h. Children and adolescents were categorized into three groups: (i) < 2 h (answers from 1 to 4); (ii) 2–4 h (answers 5 or 6); and (iii) > 4 h (answer 7). Finally, data concerning the number of TVs, computers and consoles at home, and the presence or not of these technologies in the bedroom were collected.

### Statistical analysis

The proportion (%) of children and adolescents who spent < 2 h, 2–4 h and > 4 h on each sedentary activity (TV viewing, computer games, console games and surfing the internet) was calculated separately for week and weekend days and stratified according to sex and age group. Fisher’s exact test Bonferroni’s correction was used to compare proportions by sex, age and day (weekday vs. weekend). Also, the percentage of participants who spent more than 2 h in total TV viewing, playing computer and console games, and using the internet (non-study reasons) during the whole week, during weekdays, and the weekend was calculated, and was split by age groups and sex using contingency tables. Statistical differences between groups were performed by Z-test (*T*-test when n < =30) with a 95% confidence level. Binary logistic regression analyses were performed to obtain the odds ratio (OR) adjusted by Bonferroni’s test of the different sedentary behaviors ≥2 h/day according to age (<13 years and ≥13 years), sex, having a TV, console or computer in the bedroom. All statistical analyses were run on SPSS for Windows statistical software package version 22.0 (IBM Corporation, Armonk NY, USA). The level of statistical significance was set at *p*-value < 0.05.

## Results

Table 1 shows the percentage of participants who spent more than 2 h in front of a screen; 48.4% of the total participants spent > 2 h every day of the week, 49.3% during the week and 84.0% during weekends. There were significant differences between children and adolescents for every day of the week and during weekdays (all *p* < 0.05). These differences were also observed between females and males.

**Table 1:** Percentage of participants who spend more than 2 h in sedentary behaviors by age groups and sex

	Total			Children			Adolescents		
	Total	Females	Males	Total	Females	Males	Total	Females	Males
>2 h all days	48.4	44.7	50.6	37.6	34.5	39.7	59.2*	56.8*	60.6*
>2 h week days	49.3	46.6	51.0	38.5	36.8	39.7	60.2*	58.1*	61.3*
>2 h weekend	84.0	82.6	84.8	82.2	80.5	83.3	85.8	85.1	86.1

*p < 0.05 children vs adolescents

On Tables 2 and 3, prevalence information of sedentary behaviors on weekdays and weekend days by sex and age group is presented.

**Table 2:** Percentage of participants according to time spent in sedentary behaviors by age groups and sex in total sample

		Children			Adolescents		p (age diff)		Males			Females		p (sexes diff)
	<2 h	2–4 h	> 4 h	< 2 h	2–4 h	> 4 h		< 2 h	2–4 h	> 4 h	< 2 h	2–4 h	> 4 h	
TV viewing														
Weekdays	83.1	16.4	0.5	74.9	20.7	4.4	0.048	80.1	17.8	2.2	76.3	19.9	3.8	0.428
Weekend*†	41.8	48.8	8.9	40.2	46.1	13.7	0.307	42.4	47.2	10.4	40.5	45.8	13.7	45.8
Computer Games														
Weekdays	98.6	1.4	0.0	91.0	8.0	1.0	0.005	93.4	6.1	0.4	89.8	9.4	0.8	0.345
Weekend*†	86.3	11.9	1.9	76.1	19.9	4.0	0.040	74.9	21.1	4.0	69.	26.4	4.7	0.350
Console Games														
Weekdays	98.1	1.4	0.5	94.9	4.6	0.5	0.173	95.0	4.6	0.4	93.6	6.5	0.0	0.400
Weekend*†	86.6	11.0	2.4	82.1	13.8	4.1	0.413	78.1	17.5	4.5	75.8	19.4	4.8	0.838
Internet (non-study)														
Weekdays	94.8	4.3	1.0	81.0	14.5	4.5	0.001	89.1	7.9	3.0	84.4	10.9	4.7	0.380
Weekend*†	89.2	8.0	2.4	64.2	25.4	10.5	<0.001	77.2	16.5	6.3	68.2	22.5	9.3	0.088
Internet (study reasons)														
Weekdays	96.7	1.9	0.9	94.6	4.5	1.0	0.325	96.3	3.2	0.4	95.4	3.9	0.8	0.713
Weekend	96.1	1.0	0.0	91.0	8.0	1.0	0.001	96.1	3.4	0.4	93.8	5.5	0.8	0.512
Studying (without internet)														
Weekdays	74.2	12.2	13.6	63.9	19.8	16.3	0.053	70.6	14.1	15.3	67.4	17.1	15.5	0.655
Weekend*†	78.9	20.2	0.9	77.7	18.3	4.0	0.127	78.7	20.0	1.3	77.5	20.2	2.3	0.752

Sex, age and weekdays-weekend differences using Fisher’s exact test Bonferroni’s correction

* *p* < 0.001 weekdays vs. weekend in males; † *p* < 0.001 weekdays vs weekend in females

**Table 3:** Percentage of participants according to time spent in sedentary behaviors by sex inside each age groups

	Male Children			Female Children			p (sex diff)	Male Adolescents			Female Adolescents			p (sex diff)
	< 2 h	2–4 h	> 4 h	< 2 h	2–4 h	> 4 h		< 2 h	2–4 h	> 4 h	< 2 h	2–4 h	> 4 h	
TV viewing														
Weekdays	84.9	15.1	0.0	80.5	18.4	1.1	0.383	76.3	19.8	3.8	72.2	22.2	5.6	0.760
Weekend*†	45.2	49.2	5.6	37.2	48.8	14.0	0.090	40.5	45.8	13.7	39.7	46.6	13.7	0.994
Computer Games														
Weekdays	97.6	2.4	0.0	100.0	0.0	0.0	0.152	89.8	9.4	0.8	93.1	5.6	1.4	0.588
Weekend*†	82.5	14.3	3.2	91.8	8.2	0.0	0.092	69.0	26.4	4.7	88.9	8.3	2.8	0.005
Console Games														
Weekdays	96.8	2.4	0.8	100.0	0.0	0.0	0.261	93.5	6.5	0.0	97.2	1.4	1.4	0.270
Weekend*†	81.0	15.1	4.0	95.2	4.8	0.0	0.035	75.8	19.4	4.8	93.1	4.2	2.8	0.004
Internet (non-study)														
Weekdays	95.2	4.0	0.8	94.1	4.7	1.2	0.928	84.4	10.9	4.7	75.0	20.8	4.2	0.162
Weekend*†	88.9	8.7	2.4	90.7	7.0	2.3	0.162	68.2	22.5	9.3	56.9	30.6	12.5	0.279
Internet (study reasons)														
Weekdays	97.6	2.4	0.0	96.5	1.2	2.3	0.188	95.3	3.9	0.8	93.2	5.5	1.4	0.795
Weekend	99.2	0.8	0.0	98.8	1.2	0.0	0.778	93.8	5.5	0.8	86.3	12.3	1.4	0.203
Studying (without internet)														
Weekdays	74.6	10.3	15.1	73.6	14.9	11.5	0.498	67.4	17.1	15.5	57.5	24.7	17.8	0.328
Weekend*†	80.2	19.8	0.0	77.0	20.7	2.3	0.225	77.5	20.2	2.3	78.1	15.1	6.8	0.216

Sex, age and weekdays-weekend differences using Fisher’s exact test Bonferroni’s correction

* *p* < 0.001 weekdays vs. weekend in children; † *p* < 0.001 weekdays vs weekend in adolescents

### TV viewing

The use of TV was higher on weekend days than on weekdays (*p* < 0.001) regardless of age and sex. 57.7% of children and 59.8% of adolescents watched TV for more than 2 h on weekend days. On weekdays, a higher percentage of adolescents watched TV for >2 h/day compared to children (25.1 vs 16.9%, *p* < 0.05), while on weekdays, no age difference was observed in both sex groups. On the other hand, no differences were observed between sexes on both weekdays and weekends regarding TV viewing.

### Electronic games (computer games and console games)

At weekends, the use of computer and console games was higher than on weekdays (*p* < 0.001). Adolescents played more computer games than children during both week and weekend days (*p* < 0.05). Specifically, male adolescents significantly played more computer and console games than females during weekends (*p* < 0.05). Likewise, male children played significantly more console games during weekends than females (*p* ≤ 0.01).

### Internet use for non-study reasons

At weekends, the use of internet for non-study reasons was higher than on weekdays (*p* < 0.001), as a higher percentage of both children and adolescents surfed for more than 2 h per day. The percentage of adolescents using internet for non-study reasons was higher both on weekdays and weekends (*p* < 0.001) than children. However, when splitting the age groups by sex (Table 2), no differences were observed.

### Internet for academic reasons

The use of internet for academic reasons was significantly lower in children (*p* = 0.001) than in adolescents during weekends; nevertheless, no significant differences were observed between sexes.

### Study without internet use

The reported time spent studying for less than 2 h without internet use was higher at weekends than on weekdays (*p* < 0.001). A total of 25.8% of children and 36.1% of adolescents sit for more than 2 h on weekdays, and 21.1 and 22.3%, respectively, on weekends for study reasons. However, no differences between sexes and age groups were observed on both weekdays and weekends.

### Electronic device availability at home and in the bedroom

The number of TV sets, computers, and consoles both at home and in the bedroom are depicted in Table 4. Almost all had TV and 90% had at least one computer at home. Consoles were available in 61% of homes. Males had more TVs and consoles in general at home and especially in their rooms than females (all *p* < 0.05).

**Table 4:** Percentage of participants having TVs, computers and consoles at home and in the bedroom

	At home								In the bedroom	
	0		1		2		≥3		Yes	
	Females	Males	Females	Males	Females	Males	Females	Males	Females	Males
TV	0.6	0.0	22.5	16.3	42.5	39.7	34.4	44.0	30.6	40.6*
Computer	3.8	6.2	50.9	46.3	25.2	31.5	20.1	16.0	33.6	38.6
Console	26.9	12.1*	40.6	45.9	17.5	21.0	15.0	21.0	30.8	44.4*

*Sex significant differences using Fisher’s exact test Bonferroni’s correction (* *p* < 0.05)

### Odds ratio of sedentary behavior (>2 h/day)

Table 5 shows the results of the binary logistic regression. Boys were more likely to play console and computer games >2 h/day on weekends. Compared to children, adolescents were more likely to watch TV >2 h/day on weekdays, to play computer games on weekdays and to surf on the internet for >2 h/day on weekdays and weekends and to use internet for study reasons >2 h/day on weekend days. Likewise, to have a console in the bedroom was associated with playing console and computer games >2 h/day during the weekend.

**Table 5:** Binary logistic regression analyses predicting >2 h/day of different sedentary actions

	Gender (boy)	Age (>13)	TV bedroom	Computer bedroom	Console bedroom
TV viewing >2 h					
Weekdays	1.43 (0.81–2.54)	2.40 (1.37–4.19)*	1.34 (0.71–2.52)	0.62 (0. 34–1.13)	1.04 (0.55–1.96)
Weekend	1.56 (0.96–2.55)	1.43 (0.90–2.27)	1.17 (0.69–2.00)	0.79 (0.49–1.30)	1.07 (0.63–1.81)
Total	1.66 (1.01–2.71)	1.62 (1.00–2.62)	0.94 (0.54–1.64)	0.70 (0.42–1.18)	0.99 (0.57–1.72)
Computer Games >2 h					
Weekdays	0.72 (0.22–2.36)	6.17 (1.70–22.36)*	1.56 (0.48–5.09)	0.64 (0.22–1.87)	3.01 (0.90–10.71)
Weekend	0.42 (0.21–0.81)*	1.77 (1.01–3.11)	1.36 (0.72–2.59)	1.23 (0.69–2.20)	1.93 (1.02–3.65)
Total	0.23 (0.05–1.04)	3.75 (1.29–10.85)	1.39 (0.45–4.26)	0.85 (0.32–2.27)	2.23 (0.71–7.01)
Console Games >2 h					
Weekdays	0.33 (0.07–1.657)	2.94 (0.87–9.5)	4.46 (1.09–18.20)	1.47 (0.47–4.66)	0.71 (0.291–2.66)
Weekend	0.27 (0.12–0.61)*	1.73 (0.94–3.2)	1.75 (0.88–3.50)	0.97 (0.51–1.82)	3.21 (1.59–6.50)*
Total	0.31 (0.09–1.08)	1.50 (0.62–3.66)	3.21 (1.09–9.41)	1.103 (0.41–2.57)	1.41 (0.49–4.05)
Internet (non-study) >2 h					
Weekdays	1.70 (0.83–3.46)	4.25 (2.06–9.51)*	1.84 (0.84–4.07)	1.80 (0. 89–3.62)	0.94 (0.42–2.10)
Weekend	1.764 (0.91–2.93)	4.65 (2.60–8.31)*	1.73 (0.92–3.27)	1.92 (1.09–3.38)	1.13 (0.58–2.15)
Total	1.80 (0.90–3.58)	3.94 (1.91–8.09)*	2.09 (0.97–4.549)	2.00 (1.01–4.00)	0.89 (0.41–1.94)
Internet (study reasons) >2 h					
Weekdays	1.01 (0.30–3.50)	1.70 (0.53–5.48)	0.64 (0.18–2.32)	2.30 (0.70–7.56)	3.50 (0.91–13.51)
Weekend	2.37 (0. 78–7.20)	6.71 (1.64–35.26)*	0.76 (0.22–2.71)	4.03 (1.19–13.63)	2.18 (0.61–7.78)
Total	1.18 (0.21–6.69)	5.96 (0.68–51.7)	1.31 (0. 19–8.86)	6.41 (0.73–56.68)	5.74 (0.53–62.64)
Studying (without internet) >2 h					
Weekdays	1.07 (0.65–1.78)	1.65 (1.01–2.68)	0.88 (0.50–1.55)	1.21 (0.72–2.03)	0.71 (0.40–1.25)
Weekend	1.17 (0.67–2.07)	0.95 (0.55–1.65)	1.74 (0.94–3.25)	1.33 (0.75–2.37)	0.75 (0.40–1.41)
Total	1.17 (0.67–2.01)	1.18 (0.69–2.00)	1.30 (0.71–2.39)	1.53 (0.88–2.68)	0.74 (0.40–1.37)

Data are expressed as odd ratio (95% CI)

* *p* <0.05 adjust by Bonferroni’s test

## Discussion

The present study describes the prevalence of sedentary behaviors in a representative sample of Spanish children and adolescents. Adolescents spent more time watching TV, playing with computer games, surfing on the internet (both for study and non-study reasons) than children. Except for using the internet for study reasons, for all other sedentary behaviors assessed in the ANIBES study, Spanish children and adolescents reported more screen time on weekend days than on weekdays. The only difference by sex was observed for time spent playing console and computer games, which was higher in males on weekends, the later only among adolescents. Taken together, almost 38.5% of children and 60.2% of adolescents spent > 2 h/day in front of a screen during weekdays, and 82.2 and 85.8%, respectively, during weekend days.

Given that young people have incorporated new technologies into their leisure-time activities to a great extent [30] and that there has been a decreasing trend in Spain of active commuting to school [31], moderate-vigorous activity and active commuting (mainly walking) to school should be encouraged, among other activities [32, 33]. Along the same lines, Bucksch et al. (2016) reported that the amount of screen-time behaviors among adolescents has increased in many parts of Europe and North America during the last decade [34]. In England, 40% of children aged 2 to 15 years reported being sedentary (not including TV viewing) for more than two hours per day on weekdays and 53% on weekend days [35]. Sisson et al. (2009) presented that the total proportion of young people engaged in TV/video viewing, computer use, and total screen time ≥ 2 h daily was 33.0%, 6.7%, and 47.3%, respectively. Data from Canada indicated alarming results with 80.6% reporting that they spent accumulatively a total of more than 2 h/day watching television, playing video games, and using a computer [36]. Martinez-Gómez et al. (2012), observed in 1,724 Spanish adolescents (882 girls), aged 13 to 16 years from the Region of Madrid that over 63% were not meeting the recommendation to avoid sedentary behavior on weekdays and 87% did not comply with this recommendation on weekends [37]. These data are similar to the results obtained in the present study in a representative sample for Spanish children and adolescents. In our study, 37.6% of children and 59.2% of adolescents spent > 2 h/day in front of a screen during every day of week.

On the other hand, in our study, a total of 16.9% of children and 25.1% of adolescents exceeded 2 h/day based only on their TV viewing during weekdays, while this percentage increases on weekends (57.7% of children and 59.8% of adolescents). Along the same lines, other studies have reported higher TV viewing on weekends compared with weekdays [26, 38, 39]. Among German adolescents, TV viewing on weekdays, but not on weekends, declined steadily over time with a difference between 2002 and 2010 of 12.4 min/day in girls and 18.3 min/day in boys in The Health Behaviour in School-aged Children (HBSC) [40]. Data from HBSC Spain indicated that approximately 50% of young people viewed TV >2 h/day in all age groups [41]. In agreement with other studies [26, 42], we obtained significant differences (*p* < 0.005) between sexes during weekend days for computer games in adolescents and for console games in children and adolescents. Edelson et al. (2016) observed that older children spent more time watching TV and using computers/video games than did younger children (trend across age categories for TV: *p* < 0.001 and computer: *p* < 0.001) [43]. Moreover, in our study, electronic games were predominantly chosen by males, and also, males played more than females. Female adolescents surfed the internet more than males, for both study and non-study reasons, which was in accordance with other studies [26, 44, 45]. However, Brooks et al. (2016) have observed that girls are increasingly engaged in game play [45].

New generations have an increased use of communication technologies and available information on the internet [46]. Our results showed that more than 30% had a TV, computer and/or console in their bedrooms. In addition, a total of 44% of the males reported having three or more TVs at home. Brindova et al. (2014) revealed that 70% of their study participants had TV in their bedrooms. Although Rey-López et al. (2010) observed that TV was the dominant device followed by computers and consoles in the bedroom, our results showed that the console was the dominant device used by males and the computer by females. Remarkably, only 12.1% of males and 26.9% of females didn’t have any consoles at home and these data showed significant differences between sexes. Delmas et al. (2009) observed that having a personal TV was associated with higher TV viewing [47]; however, in our study, to have TV in the bedroom was not associated with TV viewing or playing PC or console games >2 h/day on none time. On the other hand, during weekends, to have a console in the bedroom implied more time spent playing with console games (OR = 3.21; 95% CI 1.59–6.50).

Current evidence indicates that the relationship between watching TV and BMI is driven by increased energy intake, and specifically TV commercial viewing [48–51]. Epstein et al. (2008) found significant reductions in BMI in children who decreased TV viewing [48]. Another intervention study demonstrated the positive effects of the decrease of screen time among schoolchildren [52]. Young people constitute the primary target for public health strategies, which represent the possibility of health promotion and protection against chronic diseases. It is also important to take into account intervention strategies for parents, targeting parental regulation [53]. Regarding family structure (not assessed in the ANIBES study), McMillan R et al. (2015) found in their study that parental structure and child custody arrangements did not have a significant impact on screen time among youth [36].

The ANIBES study has several strengths which include the careful design, protocol, and methodology used, and was conducted among a random representative sample of the Spanish population aged 9–17 years. The validated questionnaires used to collect information on sedentary behaviors have shown good reliability and reproducibility. One limitation of this study is its cross-sectional design, which provides evidence for associations but not causal relationships. Measures of sedentary behaviors relied on self-report and could be biased, although a careful multistep quality control procedure was implemented to minimize bias. Additional limitations could be the high type I error rate due to hundreds of comparisons and secondary outcomes (also inflates type I error rate).

## Conclusions

In conclusion, a high percentage (48.4%) of children and adolescents in Spain are not meeting recommendations regarding sedentary behavior, especially and paradoxically during weekends (84.0%). Age appears as an important determinant of a sedentary lifestyle, as adolescents spent more time on screen time than children. Considering our results and the importance of reducing sedentary behaviors, efforts to reduce time spent sitting for non-study reasons are needed. The findings also suggest that the weekend may be a critical target for interventions aiming at reducing screen time because TV viewing and computer use are particularly high on weekend days. In this sense, it would be necessary to promote interesting activities in different settings (family, school, community) as alternatives to these long sitting periods during spare time.
